# The Microphone

**Published:** 1885-07

**Authors:** A. Thornburgh

**Affiliations:** Ringgold, Ga.


### THE MICROPHONE.

---

*Editors Atlanta Medical and Surgical Journal:*

Dr. Edward Joseph Eve's article in the May number of your JOURNAL, on the use of the microphone "as a diagnosticator of the physical organism," recalls to my mind some of the experiments already made with this little instrument. The Doctor's idea seems to be a good one, and the failures in the past may lead to further experiment and more favorable results. Why not?

As early as 1878, Professor J. W. Holland, of the University of Louisville, Kentucky, made some very interesting experiments with the microphone in physical diagnosis, covering the same field of thought suggested by Dr. Eve, and arrived at the following conclusions:

1. That the noisy hospital ward is a bad site for the test.

2. That success is dependent largely upon the amount of flesh that covers the ribs of the subject. The sound waves are never transmitted with clearness unless the person is lean.

3. That modifications produced by disease are not reported with a distinctness surpassing, if equal, to that attained by the unaided ear.

4. That the rythmic sounds of the heart mask all others produced in the chest, so as to make the instrument of no value in pulmonary diagnosis.

5. That a medley of sounds, like rubbing and thumping, probably due to the movements of the fœtus, utterly annihilate sounds of the fœtal heart, such as are plainly audible to the trained ear unassisted.

6. That the prospect of a successful employment of the microphone for physical diagnosis is, from present appearances, not very encouraging.—*Louisville Medical News*, *July 20th*, *1878*.

According to the above experiments, and the conclusions drawn from them, we turn away in despair of any help from this most powerful little instrument.

The value of a microphone in operations for stone and other delicate surgical manipulations has been shown at the London University College. The apparatus consists of the usual cable of wires connected with two telephones running to different parts of the city. The ordinary sound used in operations for crushing the stone was attached by a wire to the circuit of the battery. Near the handle a piece of carbon was carefully balanced and attached by a delicate spring to the battery circuit. When the end of the sound strikes against the smallest piece of calculus, the acoustic wave is transmitted along the steel of the instrument to the carbon, where it is transformed into electric vibrations, which are multiplied through the telephone, so that the noise becomes loud and unmistakable. The carbon arrangement on the sound must not be too delicate, nor the battery too strong, but with the microphone properly adjusted, it was easy by trial to detect the presence of even a minute fragment of unremoved calculus. The carbon needed only to be fitted to the probe, also, to detect bullets or fragments of bone. But while it is quite possible for a skillful surgeon to make himself absolutely certain, by means of the microphone, of what he was previously only morally convinced of, no very remarkable results, at least in ordinary practice, are anticipated from the use of the instrument.—*Journal of Materia Medica, September, 1878.*

Now, if the above be true, Dr. Eve's idea is not only practical, but already eminently successful in surgical practice, and deserving of the highest commendation as an instrument of diagnosis.

British journals state that Surgeon Mayor Wallich, of the British army, has produced an instrument, by means of which the heart sounds are not only greatly increased, but remain quite distinct, the character of the two sounds being much intensified, by applying the microphone over the stomach, one can hear the noise of water dropping into that organ from the œsophagus. There is every reason to hope that Dr. Wallich's instrument may be made available for the purpose of chemical demonstration.—*Medical and Surgical Reporter.*

Dr. Eve seems to be sanguine of a successful instrument being devised and adopted to the use of the physician and surgeon, and if Dr. Wallich's improved instrument should be further improved and simplified, so that the busy practitioner could have the benefit of this most powerful means of physical diagnosis, it would certainly be a result fraught with the most momentous consequences to the medical profession, as well as kindred arts.

The experiments of Professor Holland may not have been conducted under the most favorable auspices, and if they were, we we hope that improved instruments and more extended observations will develop grander results in this prolific field of philosophical research.

Respectfully,

A. THORNBURGH.

*Ringgold, Ga., June, 1885.*

---

VAGINAL HEMORRHAGE FOLLOWING FIRST COITUS.—Dr. Paul F. Mundé communicates notes of two most interesting cases of severe vaginal hemorrhage in newly-married women, to the Boston *Medical and Surgical Journal;* and from the rarity of the accident, and its very probable non-recognition, the paper is worthy of notice. In both cases the bleeding took place immediately after the first introduction of the male organ, and in neither did any degree of violence appear to have been used, nor was there any disproportion between the relative sizes of the parts. A rent in the left wall of the vagina was found in both women, on examination with Sims' speculum, laceration of the hymen forming its starting point. Hemorrhage was severe and continuous in both cases, and its source not having been recognized by other physicians who were called in before Dr. Mundé, and as it meanwhile resisted all treatment, the condition of the patients was greatly reduced in consequence. Dr. Mundé's method of controlling the flow of blood was to tampon the vagina tightly with discs of alum cotton, the cases subsequently doing well, and suffering no return of the inconvenience. In cases where the rent is external, where a vaginal tampon would be useless, Dr. Mundé recommends the deep suture.—*Med. Press.*

